# Dynamic risk stratification using Markov chain modelling in patients with chronic heart failure

**DOI:** 10.1002/ehf2.14028

**Published:** 2022-06-23

**Authors:** Syed Kazmi, Chandrasekhar Kambhampati, John G.F. Cleland, Joe Cuthbert, Khurram Shehzad Kazmi, Pierpaolo Pellicori, Alan S. Rigby, Andrew L. Clark

**Affiliations:** ^1^ Department of Academic Cardiology Hull University Teaching Hospital NHS Trust Hull UK; ^2^ Department of Computer Science and Technology University of Hull Hull UK; ^3^ Robertson Centre for Biostatistics and Clinical Trials University of Glasgow Glasgow UK; ^4^ Department of Cardiorespiratory Medicine, Centre for Clinical Sciences, Hull York Medical School University of Hull Hull UK; ^5^ Department of General Medicine Ghurki Trust Teaching Hospital Lahore Pakistan; ^6^ Hull York Medical School University of Hull Hull UK

**Keywords:** Heart failure, Absorbing Markov chains, Disease trajectory, Artificial intelligence, Machine learning

## Abstract

**Aims:**

Risk changes with the progression of disease and the impact of treatment. We developed a dynamic risk stratification Markov chain model using artificial intelligence in patients with chronic heart failure (CHF).

**Methods and results:**

We described the pattern of behaviour among 7496 consecutive patients assessed for suspected HF. The following mutually exclusive health states were defined and assessed every 4 months: death, hospitalization, outpatient visit, no event, and leaving the service altogether (defined as no event at any point following assessment). The observed figures at the first transition (4 months) weres 427 (6%), 1559 (21%), 2254 (30%), 1414 (19%), and 1842 (25%), respectively. The probabilities derived from the first two transitions (i.e. from baseline to 4 months and from 4 to 8 months) were used to construct the model. An example of the model's prediction is that at cycle 4, the cumulative probability of death was 14%; leaving the system, 37%; being hospitalized between 12 and 16 months, 10%; having an outpatient visit, 8%; and having no event, 31%. The corresponding observed figures were 14%, 41%, 10%, 15%, and 21%, respectively. The model predicted that during the first 2 years, a patient had a probability of dying of 0.19, and the observed value was 0.18.

**Conclusions:**

A model derived from the first 8 months of follow‐up is strongly predictive of future events in a population of patients with chronic heart failure. The course of CHF is more linear than is commonly supposed, and thus more predictable.

## Introduction

Chronic heart failure (CHF) is very common and consumes a lot of health care resource.^1^, ^2^, ^3^ Patients with CHF have a high mortality and are admitted to hospital frequently.^4^ The greatest contributor to the cost of treatment for CHF is hospitalization.^5^ The magnitude of the problem of CHF is difficult to assess with precision since there is no gold standard for the diagnosis of heart failure.^6^ Most modelling at the moment tends to be at the level of applying scoring systems to individual patients to assess the risk of death, which might be helpful for that patient, but does not describe patterns of disease behaviour at a population level.^7^, ^8^

For patients with CHF, the clinical interest lies not only in the final outcome but in the dynamics of the progress of the disease, particularly the need for hospitalization.^8^, ^9^, ^10^ Electronic data offers a way of trying to describe the trajectory of the disease course in many groups of patients.^10^, ^11^, ^12^ It might be helpful to construct a model which could describe how a group of patients might progress after an assessment for possible heart failure.^13^, ^14^, ^15^ A successful model might allow prediction at the level of the individual patient, and also would allow estimates to be made of need for health care resources to match patient need.

We therefore used Markov chains to model the progression of CHF in a well‐characterized cohort of patients referred for assessment of possible heart failure, based on a finite number of mutually *exclusive* and *exhaustive* distinct states. Markov models are applied extensively by the National Institute for Health and Care Excellence (NICE) in health economics appraisals^16^ of healthcare interventions. We take their ideas one stage further. We were particularly concerned to see if we could use events at an early stage in an individual's journey as a patient to predict what was likely to happen to them during subsequent follow‐up. This might improve understanding of the journey of the patient with CHF and allow more rational service planning and disposition of resources.

## Methods

### Ethics approval

The investigation conformed to the principles outlined in the Declaration of Helsinki. It was approved by the Hull and East Yorkshire Research Ethics Committee (Heart Care Study ELSY 2642). All subjects gave written informed consent.

### Setting

Hull is a geographically isolated area of the United Kingdom with a stable population of about 600 000 people. In this region, our hospital is the sole provider of acute medical care. In 2000, the Hull LifeLab database was established as a systematic approach to the assessment and management of people with suspected heart failure referred by physicians in primary or secondary care. Accordingly, the population includes a mixture of patients, including some who do not have heart failure by any criterion, some who have heart failure by only some criteria, as well as those with definitive evidence of heart failure. The cohort is relatively immune to further changes in diagnostic criteria for heart failure, since it does not exclude patients for whom there is diagnostic uncertainty. Patients are systematically reviewed and examined by a doctor who is a heart failure specialist. Patients are followed up at regular intervals, usually at consecutive four monthly periods. We used data from the period 2000 to 2017. The database contains information on demography, symptoms and signs, haematology and biochemistry profile (including amino‐terminal pro‐brain natriuretic peptide [NT‐proBNP]) and echocardiograms. Data are linked to the Office for National Statistic (ONS) mortality data to get the date and cause of death. We used the hospital episode statistics (HES) to determine hospital admissions at each 4‐month interval.

### Diagnostic categories and definitions

CHF was defined as the presence of signs and symptoms of the syndrome with either moderate or worse left ventricular systolic dysfunction (LVSD) (LVEF ≤40% ‐ HF with reduced ejection fraction [HeFREF]), or no or mild LVSD (LVEF >40%) and raised levels of NT‐proBNP (HF with preserved ejection fraction [HeFPEF]). NT‐proBNP ≥125 ng. L is the diagnostic threshold specified in the European Society for Cardiology (ESC)^17^ guidelines. However, the National Institute for Clinical Excellence (NICE)^16^ guidance recommends a cut‐off of NT‐proBNP ≥400 ng. L. Therefore, the population was classified into different cohorts as follows.

1. HeFREF – those with LVEF ≤40%.
2. HeFPEF – those with LVEF >40% and NT‐proBNP:
  - ≥400 ng/L
  - 125–399 ng/L
3. Controls – patients who did not fulfil criteria for cardiac dysfunction (i.e. those with LVEF >40% and NT‐proBNP <125 ng/L)
4. No NT‐proBNP – the diagnosis of HF of this group of patients was uncertain (i.e. those with LVEF >40% and no NT‐proBNP).

NT‐proBNP was not available for all patients as it was only introduced as a clinical assay during the course of the study. ‘Controls’ are referred to in inverted commas patients are not *normal* despite having normal cardiac investigations: a referring clinician thought heart failure was a possible diagnosis. Patients whose LVEF was not available at baseline (BL) have been excluded from this analysis (*n* = 143; Supporting Information, *Figure* *S1*).

### Data transformation and state definition

The states of patients were determined at consecutive four monthly intervals (cycles) after baseline. We defined the following possible states:

1. $\left[\text{Dead}\right]$ ‐ death (any cause).
2. $\left[\text{Left}\right]$ ‐ patients who left the system and had no further interaction with the service (but had *not* died or used the service for the remaining period of study).
3. $\left[\text{Hosp}\right]$ ‐ any heart failure hospitalization during the 4 month cycle (with or without a clinic visit).
4. $\left[\mathrm{OPD}\right]$ ‐ attendance for a heart failure out‐patient visit during the cycle (without either admission or death).
5. [ Noevent] ‐ a patient did not attend the service during that 4 month period, but did have a *subsequent* event, and so is not in the $\left[\text{Left}\right]$ category.

**Figure 1:**
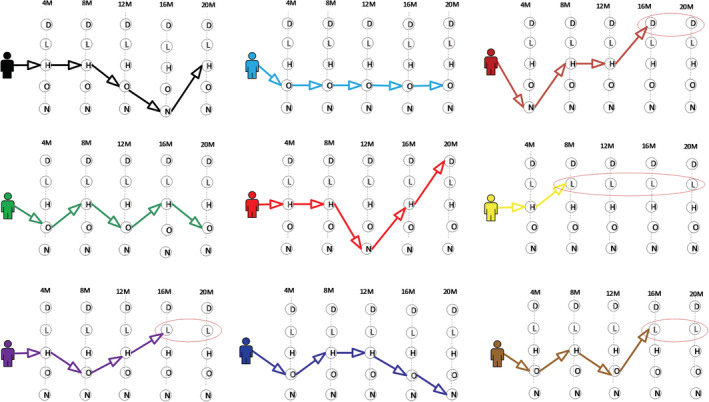
Illustrates some possible transitions for patients up to five cycles after BL (four monthly intervals). D = [*Dead*]*,* L = [*Left*], H = [*Hosp*], O [*OPD*] and N = [*No event*]. 4 M = 4 month, 8 M = 8 month, 12 M = 12 month, 16 M = 16 month and 20 M = 20 month, BL = Baseline. An oval shaped circle is drawn to show that a patient who transited to either D *or* L state will remain in this states for the rest of cycles or until a process ends.

[ Left] and [ Noevent] were treated as ‘non‐clinical’ states and were used to represent periods when the HF service was not used (*Figure* 1).

### Markov models and chains

Markov models are designed to model prognosis for clinical problems with ongoing risk after a particular event – such as hospitalization with HF.^12^, ^18^, ^19^, ^20^ The changes in a patient's health condition can be described through various distinct states ( s) (see above). Movement between n states is defined by $n^{2}$—*transition probabilities*—which determine the likelihood of patient moving from one health state within a specified time period (referred to as a ‘*cycle’*). The transition probabilities of each cycle can be represented by an n×n matrix, P, as shown in equation a:

(a) $$\left[\begin{array}{cccc} p_{1,1} & p_{1,2} & \cdots & p_{1,n}\\ p_{2,1} & p_{2,2} & \cdots & p_{2,n}\\ \vdots & \vdots & \ddots & \vdots \\ p_{n,1} & p_{n,2} & \cdots & p_{n,n} \end{array}\right]=P$$

where $p_{i,j}$ is the probability ( p) of transition from i (starting state) to j (next consecutive state). For example, in equation a $p_{1,2}$ represents the probability of transitioning from state $s_{1}$ to state $s_{2}$. Similarly, the probability of transitioning from $s_{2}$ to $s_{1}$ is given by $p_{2,1}$. Note that $p_{1,2}$ is not necessarily the same as $p_{2,1}$.

Some states, such as death, are ‘*absorbing states*’; a state from which it is impossible to transition. Thus the probability of moving to any other state is 0, and the probability of remaining in the state is always 1. ^21^, ^22^ Non‐absorbing states are called *transient states*. By including absorbing states to the regular Markov chain, the model becomes an *absorbing Markov chain* (AMC).^23^, ^24^, ^25^, ^26^ The transition probability matrix (equation b) for the absorbing Markov chain is an extended version of the regular chain:

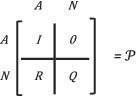

Here, the matrix can be partitioned into four separate blocks. Absorbing states (i.e. [*Dead*] and [*Left*]) precede the transient states (e.g. [*Hosp*], [*OPD*] and [*No event*]). *A* represents the absorbing states, *N* the transient states, *I* is an identity matrix and *0* is a zero matrix. *R* is a non‐zero *N*‐by‐*A* matrix and *Q* is a *N*‐by‐*N* matrix. Supporting Information, *Appendix* *S2* provides further detail.

An AMC allows an estimate to be made of the number of cycles a patient might remain in each of the transient states; and how many cycles a patient might remain in the system before reaching an absorbing state. The expected proportions or probabilities of patients entering each of the absorbing states can also be obtained. The details can be seen in Supporting Information, *Appendix* *S1*.

### Statistical analysis

Data are presented as median and interquartile range (IQR). Categorical data are presented as numbers and percentages. Differences between diagnostic groups of continuous data were tested using the independent *t*‐test. After the data transformation into health states, the distribution of patients in each state is presented in tables. All analyses were performed using R (2022.02.1), Stata software and Excel. A 2‐sided *P*‐value <0.05 was considered statistically significant.

### Data structure and time‐to‐event representation in a model

The Markov model predicts the likelihood of patient being in particular states as time passes. For example, if a patient is hospitalized, what is the probability of repeated hospitalization or transition to any other state subsequently? Transition matrices * were constructed for each of the first two transitions [between (i) baseline and end of 1st cycle (4 months), and (ii) 1st cycle and end of 2nd cycle (4 months to 8 months), respectively]. These two matrices were used to predict short‐term clinical trajectory [one‐step transition probabilities up to the 6th cycle (i.e. 24 months)] and the longer term behaviour of the system to a maximum of 4 years.

**Figure 2:**
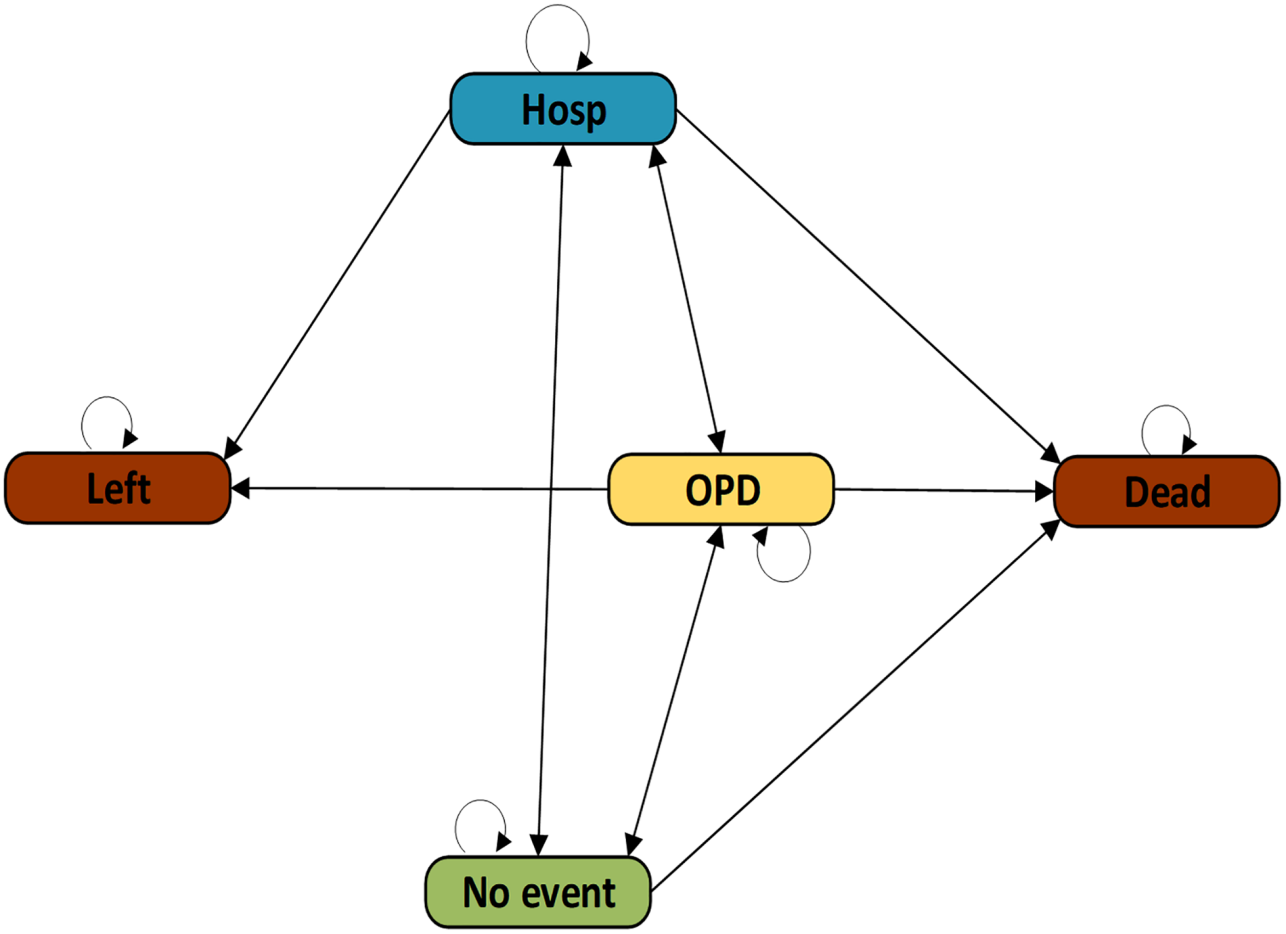
Underlying five state models for examining the disease progression among heart failure patients. The arrow indicates the directions in which instantaneous transitions are permitted. Transitions between transient states (*[Hosp]*, *[OPD]* and *[No Event]*) are potentially bidirectional, but once an absorbing state (either *[Dead]* *or* *[Left]*) has been reached, no further transitions are possible.

The underlying five state models for examining disease progression is shown in *Figure* *2*. The arrow indicates the directions in which instantaneous transitions occurred. The transitions between transient states are bidirectional, but once an absorbing state has been reached, no further transitions can be made.

Having derived a model from the observed frequencies of state transitions during the first two cycles, we applied the model to the original dataset in order to predict subsequent outcomes. We compared the probabilities of the modelled transitions with the observed transitions to see how closely the model predicted actual outcome. For the long‐term behaviour in the model as a whole, we used the modelled data to calculate a *fundamental matrix* and a *limiting matrix*. A *fundamental* matrix shows the proportion of time that an individual might spend in each of the transient states and gives an estimate of the number of cycles before a patient reaches one of the absorbing states within the lifetime of the model. A *limiting* matrix gives the expected proportions or probability of patients reaching each of the absorbing states. Details are given in appendix.

## Results

### Baseline demographics

There were 7496 patients in the study: 2620 (34%) had HeFREF, 2163 (28%) had HeFPEF (NT‐proBNP ≥400 ng/L), 1065 (14%) had HeFPEF (NT‐proBNP between 125 & 399 ng/L), 861 (11%) did not fulfil criteria for HF (‘controls’), leaving 787 (10%) with diagnostic uncertainty (normal LV systolic function but no NT‐proBNP result, ‘No NT‐proBNP’) (Supporting Information, *Figure* *S1*). Demographic and clinical characteristics of patients in each diagnostic category are shown in *Table* *1*.

**Table 1:** Baseline demographic and clinical characteristics

Variable	Missing (*n*)	Total	HeFREF	HePNEF		Control	No NT‐proBNP	*P*‐value
				≥400	125–399			
Demographics								
Age (years)		73.5 (65.6, 79.9)	72.6 (64.4, 78.9)	77.3 (71.3, 82.8)	72.8 (65.8, 79.1)	65.9 (57.7, 72.5)	74 (66.8, 80.2)	**<.001**
Men (%)		4493 (58)	1837 (74)	933 (53)	420 (48)	442 (54)	596 (47)	**<.001**
BMI (kg/m^2^)	62	28.1 (24.7, 32.1)	27.2 (24, 30.9)	28.2(24.7, 32.4)	29.6 (26, 33.5)	29.2 (26.1, 33.7)	28.6 (25.1, 32.5)	**<.001**
Underweight (<20) (%)		281 (4)	118 (5)	73 (4)	14 (2)	11 (1)	31 (3)	**<.001**
Lean (20–24.9) (%)		1723 (23)	677 (27)	379 (22)	135 (16)	132 (16)	267 (22)	
Overweight (25–29.9) (%)		2751 (36)	914 (37)	635 (37)	304 (35)	301 (37)	436 (35)	
Obese (30–39.9) (%)		2401 (32)	691(28)	531 (31)	339(39)	304(37)	428 (35)	
Morbidly Obese (≥40) (%)		421 (6)	71 (3)	119 (7)	74 (9)	63 (8)	72 (6)	
NYHA class (%)	189							
I		1936 (26)	346 (14)	362 (21)	311 (37)	378 (50)	425 (35)	**<0.001**
II		3379 (45)	1174 (47)	835 (48)	398 (47)	277(36)	542 (45)	
III		1986 (27)	886 (36)	513 (29)	130 (15)	101 (13)	230 (19)	
IV		149 (2)	75 (3)	31 (2)	6 (1)	5 (1)	13 (1)	
Systolic BP (mmHg)	24	139 (122, 158)	129 (113, 145)	142 (126, 162)	150 (133, 166)	143 (129, 158)	148 (130, 165)	**<0.001**
Oedema	686							
None		4087 (59)	1244 (55)	753 (46)	528 (65)	578 (78)	736 (65)	**<0.001**
Trace		944 (14)	343 (15)	241 (15)	120 (15)	62 (8)	135 (12)	
Ankles		1398 (20)	472 (21)	471 (29)	126 (15)	79 (11)	170 (15)	
Above ankles		524(8)	185(8)	160(10)	41(5)	21(3)	84(7)	
Left ventricular systolic dysfunction								
LV impairment	1							
None		3194 (45)	0 (0)	969 (56)	626 (72)	697 (86)	902 (72)	
Trivial		560 (8)	0 (0)	265 (15)	94 (11)	60 (7)	141 (11)	
Mild		931 (13)	0 (0)	512 (29)	147 (17)	58 (7)	214 (17)	
Worse		2485 (35)	2485 (100)	0 (0)	0 (0)	0 (0)	0 (0)	
EF Simpsons (%)	3506	47 (37, 58)	33 (26,38)	53 (47,60)	57 (50,62)	59 (54,64)	55 (48,61)	
Findings on electrocardiogram								
Heart rate (b.p.m.)	209	72 (62, 84)	75 (64, 88)	72 (62, 85)	67 (58, 78)	71 (62, 81)	71 (62, 82)	**<0.001**
Heart rhythm (Sinus) (%)	190	4976 (67)	1517 (62)	785 (45)	783 (91)	778 (97)	869 (72)	**<0.001**
QRS (ms)	356	98 (86, 118)	114 (98, 144)	96 (86, 112)	92 (82, 100)	90 (82, 98)	92 (84, 114)	<0.001
Blood test								
Haemoglobin (g/dL)	1397	13.4 (12.2, 14.5)	13.5 (12.2, 14.6)	12.9 (11.7, 14.1)	13.5 (12.6, 14.4)	14 (13.2, 15)	13.3 (12.1, 14.4)	**<0.001**
Sodium (mmol/L)	1229	139 (137, 140)	139 (136, 140)	139 (137, 140)	139 (137, 141)	139 (138, 141)	139 (137, 140)	**<0.001**
Potassium (mmol/L)	1270	4.3 (4, 4.6)	4.4 (4.1, 4.7)	4.3 (4,4.7)	4.3 (4.1, 4.6)	4.3 (4,4.5)	4.3 (4, 4.6)	0.39
Urea (mmol/L)	1229	6.5 (5, 8.9)	7.2 (5.4, 10.1)	7.1 (5.4, 9.8)	5.9 (4.7, 7.3)	5.2 (4.2, 60.3)	6.2 (4.8, 8.2)	**<0.001**
Creatinine (μmol/L	1237	97 (80, 121)	106 (88, 135)	100 (82, 129)	87 (74, 103)	82 (71, 96)	92 (77, 114)	**<0.001**
Albumin (g/L)	1331	38 (35, 40)	38 (35,40)	37 (35, 39)	39 (37,41)	40 (38, 42)	38 (35,40)	**<0.001**
NT‐proBNP (ng/L)	1116	792 (219, 2165)	1752 (745, 3900)	1269 (727, 2364)	225 (165, 296)	60 (37,91)		
Heart failure medication								
Loop diuretic (%)		4568 (60)	1975 (79)	1204 (69)	337 (39)	209 (26)	554 (44)	**<0.001**
Furosemide EquivDailyDose (mg)		40 (40, 80)	40 (40,80)	40 (40,80)	40 (40,40)	40 (40,40)	40 (40, 80)	**<0.001**
Thiazide (%)		561 (7)	107 (4)	136 (8)	104 (12)	94 (12)	102 (8)	**<0.001**
Beta‐blocker (%)		4333 (57)	1875 (75)	1077 (62)	407 (47)	211 (26)	499 (40)	**<0.001**
ACE/ARB (%)		5166 (68)	2185 (88)	1200 (69)	501 (58)	352 (43)	650 (52)	**<0.001**
MRA (%)		1584 (21)	981 (39)	302 (17)	65 (8)	27 (3)	89 (7)	**<0.001**

Continuous variables are presented as median (interquartile range), whereas categorical variables are expressed as numbers and percentage. *P*‐values are for differences between patients with HeFREF, HeFPEF, Control, and those with No NT‐proBNP recorded (calculated from analysis of variance). NT‐proBNP only became a clinical service during the course of the data collection.

BMI, body mass index (calculated as the weight in kilograms divided by height in meters squared); NYHA, New York Heart Association. BP, blood pressure; LV, left ventricular; EF, ejection fraction; LVSD, left ventricular systolic dysfunction; NT‐proBNP, N‐terminal pro–brain natriuretic peptide; ACE, angiotensin‐converting enzyme inhibitor; ARB, angiotensin receptor blocker; MRA, mineralocorticoid antagonist; ECG, electrocardiography; eGFR, estimated glomerular filtration rate; HF, heart failure; HeFREF, HF with preserved ejection fraction, HeFPEF, HF with preserved left ventricular ejection fraction (type 1 is defined as echocardiographic abnormalities that could account for symptoms and NT‐proBNP concentration >400 pg/mL, and type 2 is defined as no LVSD but NT‐proBNP concentration >400 pg/mL); Control; No NT‐proBNP, IQR, interquartile range; Bold indicates significance at the 0.05 level (two tails).

The distribution and proportion of patients following the first transition (between baseline and end of 1st cycle) are shown in *table* *2*. Table 4a of the Supporting Information, *Appendix* *S2* provides the observed frequencies following the second transition (between 1st and end of 2nd cycle) and the corresponding transition probabilities are shown in Table 4b of the Supporting Information, *Appendix* *S2*.

**Table 2:** The first transition cycle (observed from the data)

1st cycle (*N* at baseline 7496)		Absorbing states		Transient states		
		*[Dead]*	*[Left]*	*[Hosp]*	*[OPD]*	*[No Event]*
Initial	Distribution	427	1842	1559	2254	1414
	Probability	0.06	0.25	0.21	0.3	0.19

The distribution and proportion of patients following the first transition (between baseline and end of 1st cycle): Patients all started in the [OPD] state, and then over the subsequent 4 months transitioned through the five possible states with the distribution and probabilities given: 427 (6%) had died, 1842 (25%) had left the service, 1559 (21%) were admitted to hospital, 2254 (30%) had attended the out‐patient clinic, and 1414 (19%) had not accessed the service.

*N*, total number of patients; [Hosp], hospitalized; [OPD], out‐patient clinic visit.

### Model

The corresponding transition probabilities (*P*_*obs*_) seen in Table 4b of the Supporting Information, *Appendix* *S2* can be represented with four block matrices, *I*, *0*, *R* and *Q*:

(c) $$I=\left[\begin{array}{ccc} 1 & 0 & 0\\ 0 & 1 & 0\\ 0 & 0 & 1 \end{array}\right],0=\left[\begin{array}{ccc} 0 & 0 & 0\\ 0 & 0 & 0\\ 0 & 0 & 0 \end{array}\right],R=\left[\begin{array}{cc} 0.07 & 0.17\\ 0.02 & 0.18\\ 0.05 & 0 \end{array}\right]\;\text{and}\;Q=\left[\begin{array}{ccc} 0.24 & 0.14 & 0.38\\ 0.12 & 0.22 & 0.55\\ 0.19 & 0.13 & 0.63 \end{array}\right]$$

(d) $$F=\begin{array}{cc} & \begin{array}{ccc} \left[\text{H}\right] & \;\left[O\right] & \;\;\left[N\right] \end{array}\\ \begin{array}{c} \left[\text{H}\right]\\ \left[O\right]\\ \left[N\right] \end{array} & \left[\begin{array}{ccc} 2.65 & 1.24 & 4.56\\ 1.82 & 2.55 & 5.66\\ 2 & 1.53 & 7.02 \end{array}\right] \end{array}=\begin{array}{c} \text{Total}\;\text{cycles}\\ \left[\begin{array}{c} 8.45\\ 10.03\\ 10.55 \end{array}\right] \end{array}$$

From the block matrices, the *fundamental matrix* $\left(F\right)$ was obtained. D = [Dead], L = [Left], H = [Hosp], O [OPD] and N = [No event]

The F matrix (equation d) gives the expected number of visits to each non‐absorbing states before absorption. For example, the first row indicates that if the patient is in the $\left[\text{Hosp}\right]$ (*H*) state after their initial transition, then on average he/she will be in this state for approximately three cycles, in the $\left[\mathrm{OPD}\right]$ (*O*) state for one cycle and will not require HF services for five cycles (prior to reaching an absorbing state). Similarly, the second row of the matrix shows that if the patient is in the $\left[\mathrm{OPD}\right]$(*O*) state after their initial transition, then on average he/she will be expected to spend two, three, and six cycles in the $\left[\text{Hosp}\right]$(*H*), $\left[\mathrm{OPD}\right]$ (*O*) and $\left[\mathrm{No}\;\text{Event}\right]$(*N*) states, respectively. The F matrix also gives an indication of the number of cycles before a patient reaches an absorbing state, obtained by summing each row of F, shown as the final column in equation d.

The *limiting matrix* ( $\bar{P}$) (equation e) shows the probabilities of patients reaching one of the two absorbing states (death or the end of the model) as a function of the state reached after the end of the first cycle. D = [Dead], L = [Left], H = [Hosp], O [OPD] and N = [No event].

(e) $$\bar{P}=\begin{array}{cc} & \begin{array}{ccccc} \left[\text{D}\right] & \left[\text{L}\right] & \left[\text{H}\right] & \left[O\right] & \left[N\right] \end{array}\\ \begin{array}{c} \left[\text{D}\right]\\ \left[\text{L}\right]\\ \left[\text{H}\right]\\ \left[O\right]\\ \left[N\right] \end{array} & \left[\begin{array}{ccccc} 1 & 0 & 0 & 0 & 0\\ 0 & 1 & 0 & 0 & 0\\ 0.43 & 0.57 & 0 & 0 & 0\\ 0.46 & 0.54 & 0 & 0 & 0\\ 0.51 & 0.49 & 0 & 0 & 0 \end{array}\right] \end{array}$$

Equations d and e need to be read together: an example of how to interpret the information is to say that the model predicts that a patient who has been hospitalized after the first cycle has a probability of dying (*D*) of 0.43 *within (approximately) eight cycles* (where eight cycles is equivalent to an additional six cycles after the first two used the generate the model—in other words, an additional 24 months). Note that by definition, every patient has to reach an absorbing state within the timeframe of the model.

### Applying the model

*Table* *3* shows observed and predicted probabilities of transitions up to the 6th transition (2 years) for all patients. Note that the predicted probabilities derive from the model using only the data observed for the first two transitions. The table also shows the degree to which the model diverges from the reality of the observed data. The agreement for the first two cycles is necessarily identical. However, the agreement for the important clinical states, death and hospitalization, remains very strong up to 2 years from the initial assessment.

**Table 3:** Predicted and observed probabilities up to the 6th cycle (2 years) for overall population

From		Prediction (model)					Observed (from data)					Error (E) between prediction				
	Cycle	*[Dead]*	*[Left]*	*[Hosp]*	*[OPD]*	*[NE]*	*[Dead]*	*[Left]*	*[Hosp]*	*[OPD]*	*[NE]*	*[Dead]*	*[Left]*	*[Hosp]*	*[OPD]*	*[NE]*
BL	1	‐	‐	‐	‐	‐	0.06	0.25	0.21	0.30	0.19	‐	‐	‐	‐	‐
	2	‐	‐	‐	‐	‐	0.09	0.31	0.12	0.12	0.36	‐	‐	‐	‐	‐
	3	0.12	0.34	0.11	0.09	0.34	0.11	0.34	0.11	0.26	0.18	0.00	0.00	0.00	−0.17	0.16
	4	0.14	0.37	0.10	0.08	0.31	0.14	0.41	0.10	0.15	0.21	0.01	−0.04	0.01	−0.07	0.10
	5	0.16	0.39	0.09	0.07	0.28	0.16	0.47	0.10	0.07	0.20	0.00	−0.08	0.00	0.00	0.07
	6	0.19	0.41	0.08	0.07	0.25	0.18	0.54	0.08	0.19	0.00	0.00	−0.13	0.00	−0.13	0.25

The predicted probabilities derive from the model using only the observed data for the first two cycles. Note that because the model is constructed from the first two cycles, it makes no prediction for those cycles. The left‐hand columns show the predicted probabilities of the model, the columns at the centre represent the observed probabilities, and right‐hand columns show the error (E) between two. For example, at cycle 4, the model predicts 14% patients will be dead, 37% have discharged, 10% patient will be hospitalized, 08% will be attended out‐patient and 31% will not be required any HF service. Colour coding (heat map), as the difference increase changes from green to red. Negative signs indicate underestimation vice versa for positive sign. Probabilities were rounded to 2 decimal points.

Hosp, hospitalized; OPD, out‐patient clinic visit; NE, No event; BL, Baseline.

### Prediction based on demographics

To assess whether there is a difference in the progression of patents of different sex and age‐groups [≥65, <65 (years)], an AMC was developed for each sub‐group. The model continued to predict death and hospitalization with precision. The fundamental and limiting matrices (long‐term prediction) for the subgroups are shown in Supporting Information, *Appendix* *S2* (equation i to iv); women spend fewer cycles in the transient states than men, and are less likely to die than men. Similarly, younger patients had a better prognosis. Observed and predicted probabilities of transitions up to the 6th transition (2 years) for sub‐groups are shown in Tables 5a–d in the Supporting Information, *Appendix* *S2*.

**Figure 3:**
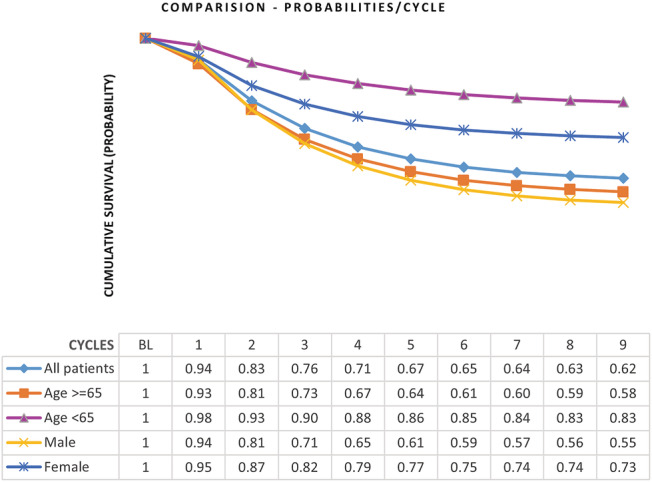
Illustrates the estimated survival probabilities for patients in the study over nine cycles. The figure shows not only survival of overall patients but also those of different age and sex subgroups. Young and women had a better survival at each cycle, however, the survival of both, the male and older patients (aged ≥ 65) sub‐groups show similar trend up‐to the 3^rd^ cycle (i.e. 12 months). It is only after this that the two sub‐groups diverge, with male have marginally better survival rate. Abbreviations: n, number of patients in each state; FU, Follow‐up; [Hosp], hospitalized; [OPD], out‐ patient clinic visit.

The probability of survival over nine cycles is illustrated in *Figure* *3*. The figure shows not only overall survival, but also survival in the different subgroups. The young and women had better survival at each cycle.

## Discussion

Dynamic risk stratification gives a different view from traditional models of disease progression by treating the clinical trajectory of patients as a group rather than by trying to predict individual survival.^12^ For example, current palliative performance scales (PPS) might be applied to individual patients to assess their risk of death within a particular timeframe (which might be helpful for that patient), but does not describe patterns of disease behaviour at a population level. Dynamic risk stratification using absorbing Markov chains in unselected patients attending a community heart failure clinic is complex and ambitious. Technically complex because it requires rigid categorization of patient's progression into a finite number of mutually exclusive and exhaustive disease states. Ambitious because it has to include two considerations. Firstly, there is no gold standard for the diagnosis of HF; secondly, the present HF scoring systems do not describe patterns of disease at a population level.

We have used a large, epidemiologically representative, database to develop a dynamic risk stratification model for patients referred for the assessment of possible heart failure to a specialist community clinic. The most striking finding is that the events which occurred during the first two cycles (i.e. over the first 8 months of follow up) allowed us to construct a model predicting future events which corresponded extremely closely to the actual, observed, events. This was particularly true for the most important clinical events we considered, namely heart failure hospitalization and death.

CHF disease management generally focuses on high‐risk patients. Such an approach can lead to reductions in hospitalizations and mortality by targeting interventions on those most at risk.^28^ However, the effectiveness of such programs over time for lower‐risk patients is uncertain. Improving the management of patients with CHF across the spectrum of risk could yield significant health gains in the longer term. Our findings are consistent with Krajewska *et al*.^14^ who reported that patients who were hospitalized in the initial 4 months will be expected to spend more cycles in the hospital when compared with other patients. Krajewska *et al*.^14^ and Zhang *et al*.^15^ emphasize that re‐evaluating risk is an important aspect of care as risk changes with time. The most recent state is a better predictor of future states than is an initial state at some remote time. Our results are subtly different in emphasis: we have found that a model derived from the first two transitions, when applied at each subsequent transition, continues accurately to predict outcome. Although risk changes as a consequence of the present state the patient happens to be in, the consequences of being in a particular state remain constant. The system has no memory—the risk is dependent upon the present state only, and not how that state was reached.

Our Markov model provides a more convenient and less computationally complex strategy than complex scoring systems to estimate the probability of transitions to and from particular states. However, as is the case in all data driven approaches, pre‐processing of the data based on (i) a knowledge of the likely clinical course and (ii) appropriately defined states is essential. Only then will the output from the model allow an understanding of transitions, and, potentially, better management of patients.

We have outlined the potential value of a model that provides a prediction of a complex problem with low computational overhead. It might be helpful not only in predicting risk states for patients but also in the allocation of resources. We studied patients across the whole spectrum of risk, which makes our study more epidemiologically representative than many multicentre studies that enrol patients in a selective and non‐consecutive fashion. The approach we have taken is practically validated by Chan *et al*.^28^ We need to verify whether the same model can be used for other datasets without training and learning. The model might then be developed further to personalize predictions.

### Limitations

The data used is from a single centre with a population of people who were referred for assessment of possible heart failure. Whilst our results indicates that AMC modelling is applicable to patients in Hull LifeLab, we cannot know if the results are more generally applicable. Importantly, the same methodology can be applied to other populations of people with heart failure. There could be errors in the coding of data. However, the errors in our predictive model were low. We have only considered a limited number of subgroups (age and sex) and not others based on other clinical variables such as NT‐proBNP.

## Conclusions

Our finding, which events early in the course of follow up allow a very strong prediction of subsequent outcomes, have important implications for understanding the trajectory of heart failure. Heart failure is often thought of as a disease with a steady downward course, punctuated by essentially unpredictable hospitalization and with an ever‐present risk of sudden death.^27^ However, our findings strongly suggest that the true course of heart failure is more linear than is commonly supposed, and thus much more predictable.

## Funding

All other authors reports none. Except Prof. Cleland is supported by a British Heart Foundation Centre of Research Excellence award RE/18/6/34217.

## Conflict of interest

All other authors declare no relationship to industry and no conflict of interest. Except Prof. Cleland has received research support and personal honoraria from Abbott, Bayer, Boehringer Ingelheim, Bristol Myers Squibb, Novartis and Vifor Pharma unrelated to the current manuscript.

## Supporting information




**Appendix S1.** Supporting Information.

**Appendix S2.** Supporting Information.

## Footnotes

* Strictly, the first transition is a *vector*: matrices have equal numbers of rows and columns. In the current model, all subjects enter in the [*OPD*] state, and thus, the first transition is a single line only.
