# Supplementary material for: Dynamic risk stratification using Markov chain modelling in patients with chronic heart failure
Source: ESC Heart Fail. 2022 Jun 23;9(5):3009–18. doi: 10.1002/ehf2.14028 (PMC9715820; doi:10.1002/ehf2.14028)
Supplement: Supplementary file 1 — Appendix S1. Supporting Information. [file EHF2-9-3009-s001.docx]

***Appendix A:***

***Markov Chain***

Any process evolving over time with uncertainty is a *stochastic process,* and models based on such processes are stochastic or probabilistic models (1, 2). Specially, the event based progression and encountering other complexities of a disease can be represented using multistate models (3). These are often based on the use of first-order Markov processes (chain, and hidden), and allow for the risk to evolve dynamically (4). If the process is both stochastic and the behavior of the model in one time period does not depend on the previous time period, the process is *Markovian, hence:*

- The process has “lack of memory”
- Even processes where the previous state does matter can be made Markovian through definition of temporary states know as *tunnel states.*

Such modelling is more applicable when interest lies in estimating the probability of transition from one state to another within a specified time period, or estimating the average period of a single stay in a state ( mean sojourn time) (5, 6). The Markov model assumes that the patient can be in any one of the many states referred to as ‘*Markov states*’ and that there are the ‘events’ which allow the transition from one state to another within a specified time period known as a “*Markov cycle”.* The memory-less property allows the model to be described in term of single-cycle transition matrix. (7) provided a regular Markov chain (RMC) functions and steps that are used to governed by transition, where:

A *stochastic process {X_n_}* is a Markov Chain if for all times $n\geq0$ and all states $i_{0},\cdots,i,j \in S$*,*

|  | $\mathcal{P}\left( X_{n+1}=j \vert X_{n}=i, X_{n-1}=i_{n-1,},\ldots,X_{0}=i_{0} \right)\mathcal{= P}\left( X_{n+1}=j \vert X_{n}=i, \right) =p_{ij} \forall i\leq n, j \leq n$ | (a) |
| --- | --- | --- |

$\mathcal{P}_{ij}$ is the probability that the chain when in state $i$, moves to the next state $j$ one unit of time later, and is often referred to as a “*one-step transition probability*”. The square matrix

|  | $P =\mathcal{(P}_{ij}), i,j\in S$ | (b) |
| --- | --- | --- |

is called a one-step transition matrix. And since leaving state $i$, the chain must move to one of the $j$ states, each row must sum to 1, i.e.,

|  | $\sum_{j\in S} \mathcal{P}_{ij}=1$ | (c) |
| --- | --- | --- |

If we make an assumption that the transition probabilities do not depend on “$n$” (the time), then using *n=0* in (a) gives

| $\mathcal{P}_{ij}= P\left( X_{1}=j \vert X_{0}=i \right)$ | (d) |
| --- | --- |

The key property of the chain is, that the next future state is dependent given the present state irrespective of past state. Therefore if $n$ is the present time, then the future is given by $\{X_{n+1}, X_{n+2}, X_{n+3}\ldots.X_{n+m}\}$ while the past of the chain is given by $\{X_{0}, X_{1}\ldots.X_{n-1}\}$ and current state is $\{X_{n}\}$. The matrix containing$\mathcal{P}_{ij}$, the transition probabilities of $n$ states, can be represented as a $n\times n$ matrix ($P$) as shown below.

|  | $P=\left[ \begin{matrix} p_{11} & p_{12} & \cdots& p_{1r} \\ p_{21} & p_{22} & \cdots& p_{2r} \\ \vdots& \vdots& \ddots& \vdots\\ p_{r1} & p_{r2} & \cdots& p_{rr} \end{matrix} \right]$ | (e) |
| --- | --- | --- |

A regular Markov Chain models have two important properties: *irreducibility and aperiodicity*. Looking at the 2 states’ (i.e., $i$ and $j$) model, if state $j$ is $accessible$ from state $i$, $i \to j$, if $P_{ij}^{n}>0$ for some $n\geq0$. This means there is a possibility of reaching $j$ from $i$ in some number of steps. If $j$ is not $accessible$ from$i$, $P_{ij}^{n}=0$ for all $n \geq0$, and thus the chain started from $i$ never visits $j$. Whereas, if $i$ is accessible from$j$, and $j$ is accessible from $i$ then this means $i$ and $j communicates$ with each other and can be represented by $i \leftrightarrow j$. The accessibility relation divides states into $classes$. Within each class, all states communicate to each other, but no pair of states in different $classes$ communicates. A regular Markov chain is irreducible if all states belong to one class, so that all states communicate with each other. For example, if the chain has $n$ states, irreducibility means that entries of $I + P + P \ldots+ P^{n}$are nonzero. Since$0<P_{ij}^{n}<1$, for irreducible states the equation (1) can also be expressed as:

|  | 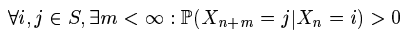 | (f) |
| --- | --- | --- |

Also, $irreducibility$ property of the chain force either all of the states are $transient$ or all are $recurrent$. A state $i$ is said to be $transient$if,

- upon entering state$i$, there is a positive probability that the process may never return to state $i$ again.
- there exists a state $j$ (different from$i$) that is accessible from state $i$ but $i$ is not accessible from $j$.
- In a finite-state Markov chain, transient state is visited only a finite number of times.

A state $i$ is said to be $recurrent$ if,

- Upon entering state$i$, the process will definitely return to state $i$.
- Since a $recurrent$ state definitely will be revisited after each visit, it will be visited infinitely often.

In the case of aperiodicity, there is no return to the state $i$ after entering to state $j$, so at this point the state $j$ have started keeping the communication to themselves only. For example, when a research is interested in: to compute the probability that Markov chain is eventually absorbed in state $i$. Secondly, to check the behaviour of the Markov chain until absorption. For example the average time spend in any other state $j \neq i$ before being stuck in state $i$. Such limitation of a regular Markov can be handle by a special type of state, one which when the process enters it, it never leaves it. Such states are referred to as $absorbing states$.

***Absorbing Markov Chain***

In an absorbing chain modelling (ACM), the terminal or censored states are fixed points or steady states once patient entered in one of these states, there is no exit from it (8). For example, a certain end points (i.e., death) or when an individual who never reaches an absorbing state (i.e., alive if the study ends) (right-censored) - whether because the study observation is ongoing or the subject has withdrawn or been lost to follow-up or either permanent or temporary immunity from a decease. The basic algorithmic structure of absorbing Markov chains (AMC) is:

*When a chain enters a state* $X_{A}$ *and remains in that state forall time,*

|  | $P\left( X_{1}=j \vert X_{0}=i \right)= \mathcal{P}_{00}=1$ | (g) |
| --- | --- | --- |

*Thus* $X_{A}$ *is called an absorbing state*

However, it is not sufficient for a Markov chain to contain an absorbing state (one or more) in order for it to be an absorbing Markov chain. It must also have all other states reach an absorbing state/s with probability 1 (equation 8). In multi-state models for intermittently-observed processes, the times of changes of state are usually interval censored, known to be within bounded or fixed-time intervals. Then a new transition matrix is determined, for the transition from the $j_{th}$ cycle to the $k_{th}$ cycle, and is given by: $P_{j}=P_{i}^{2}$. Suppose a Markov chain has initial probability vector $X_{o}=[i_{1}, i_{2},i_{3}\cdot\cdot\cdot\cdot\cdot i_{n}]$ and transition matrix $P$ then the probability vector after $n$ cycle of the analysis is

|  | $X_{0}= P^{n}$ | (h) |
| --- | --- | --- |

If the probabilities in every cycle are the same (i.e., steady state), then

|  | $P_{n}=P_{1}^{n}$ | (i) |
| --- | --- | --- |

However, it is important to note that:

- Steady-state predictions are never achieved in actuality due to a combination of
- errors in estimating *P*
- changes in $P$ over time
- changes in the nature of dependence relationships among the states.
- Nevertheless, the use of steady-state values is an important diagnostic tool for the decision maker.
- Steady-state probabilities might not exist unless the Markov chain is ergodic

The system can be modelled as an AMC by modifying the transition matrix $(P)$ of RMC into a canonical form with four block matrices, $I, 0, R$ and $Q$, as shown:

|  | **A** | \| **N** \| \| --- \| | \|  \| \| --- \| |
| --- | --- | --- | --- | --- | --- |
| **A** | **I** | **O** |  |
| **N** | **R** | **Q** |  |

Where *A* represent the absorbing states, *N* the non-absorbing states, *I* is an identity matrix and *0* is a zero matrix

**Reference List**

1. Briggs A, Sculpher M. An introduction to Markov modelling for economic evaluation. PharmacoEconomics. 1998;13(4):397-409.

2. Briggs AH, Ades AE, Price MJ. Probabilistic sensitivity analysis for decision trees with multiple branches: use of the Dirichlet distribution in a Bayesian framework. Med Decis Making. 2003;23(4):341-50.

3. Jackson CH. Multi-State Models for Panel Data: The msm Package for R. J Stat Softw. 2011;38(8):1-28.

4. Olariu E, Cadwell KK, Hancock E, Trueman D, Chevrou-Severac H. Current recommendations on the estimation of transition probabilities in Markov cohort models for use in health care decision-making: a targeted literature review. Clinicoeconomic Outc. 2017;9:537-46.

5. Sonnenberg FA, Beck JR. Markov models in medical decision making: a practical guide. Medical decision making : an international journal of the Society for Medical Decision Making. 1993;13(4):322-38.

6. Sato RC, Zouain DM. Markov Models in health care. Einstein (Sao Paulo). 2010;8(3):376-9.

7. Twumasi C, Asiedu L, Nortey ENN. Markov Chain Modeling of HIV, Tuberculosis, and Hepatitis B Transmission in Ghana. Interdiscip Perspect Infect Dis. 2019;2019:9362492.

8. Montgomery J. Absorbing Markov Chains: Department of Sociology, University of Wisconsin - Madison; 2009. 14 p.
