# Supplementary material for: Dynamic risk stratification using Markov chain modelling in patients with chronic heart failure
Source: ESC Heart Fail. 2022 Jun 23;9(5):3009–18. doi: 10.1002/ehf2.14028 (PMC9715820; doi:10.1002/ehf2.14028)
Supplement: Supplementary file 2 — Appendix S2. Supporting Information. [file EHF2-9-3009-s002.docx]

***Appendix B:***

| **Table 4 (a):** Transition frequencies for 2^nd^ cycle (observed from the data) | | | | | | | |
| --- | --- | --- | --- | --- | --- | --- | --- |
| States | | **To** | | | | | **Total** |
|  |  | ***[Dead]*** | ***[Left]*** | ***[Hosp]*** | ***[OPD]*** | ***[No Event]*** |  |
| **From** | ***[Dead]*** | 427 |  |  |  |  | 427 |
|  | ***[Left]*** |  | 1842 |  |  |  | 1842 |
|  | ***[Hosp]*** | 104 | 266 | 376 | 223 | 590 | 1559 |
|  | ***[OPD]*** | 54 | 188 | 273 | 493 | 1246 | 2254 |
|  | ***[No Event]*** | 66 |  | 269 | 190 | 889 | 1414 |
| The State-to-state distribution of patients between 1st and end of 2nd cycle: Patients in [Dead] (n=427) and [Left] (n=1842) states at the end of 1st cycle remain in these states at the next follow-up. Of 1,559 hospitalised patients, 104 patients died, 266 left the service, 376 were hospitalised again, 233 were seen in out-patients and 328 did not attend the service.  Abbreviations: n, number of patients in each state; FU, Follow-up; [Hosp], hospitalised; [OPD], out-patient clinic visit; | | | | | | | |

| **Table 4 (b):** Transition probabilities during the 2nd cycle (from 4 to 8 months FU) | | | | | | | | |
| --- | --- | --- | --- | --- | --- | --- | --- | --- |
|  |  | $I$ |  |  |  |  |  |  |
|  |  |  |  |  |  |  | $0$ |  |
|  |  |  |  |  |  |  |  |  |
| $\boldsymbol{P}_{\boldsymbol{obs}}$ **=**   \|  \| \| --- \| \| |  |  | **To** | | | | |  |
|  |  |  | *[Dead]* | *[Left]* | *[Hosp]* | *[OPD]* | *[No Event]* |  |
|  | **From** | *[Dead]* | 1 | 0 | 0 | 0 | 0 |  |
|  |  | *[Left]* | 0 | 1 | 0 | 0 | 0 |  |
|  |  | *[Hosp]* | 0.07 | 0.17 | 0.24 | 0.14 | 0.38 |  |
|  |  | *[OPD]* | 0.02 | 0.18 | 0.12 | 0.22 | 0.55 |  |
|  |  | *[No Event]* | 0.05 | 0 | 0.19 | 0.13 | 0.63 |  |
|  |  |  |  |  |  |  |  |  |
|  |  |  |  |  |  |  |  |  |
|  |  | $R$ |  |  |  |  | $Q$ |  |
|  |  |  |  |  |  |  |  |  |
| \| A 5×5 matrix, represents the observed probabilities **(** $\boldsymbol{P}_{\boldsymbol{obs}}$**)** from data at 2nd cycle.  The states are ordered such that absorbing states (**ABS**) come first and then the transient states (**TR**). The different colours represent the canonical form of our Absorbing Markov chains with four block $\boldsymbol{I, O, R and Q}$ matrices.   *Patients who died or left the system in the 1st cycle remain in those states with probability 1. As examples of transitions between transient states, patients in the [Hosp] state had a probability of being [Dead] of 0.07; and patients in the [OPD] state had a probability of being [Hosp] of 0.12.  *Description of each block matrices can be seen in appendix 1a. \| \| --- \| | | | | | | | | |

The fundamental ($F$) and limiting matrices ($\bar{P}$) showing the long-term prediction for the subgroups (sex and age-groups): for male ($m$) it is equation i, for female ($f$) it is equation ii, for age-group ≥ 65 it is equation iii and < 65 it is equation iv.

|  | $\begin{matrix} [Dead] & [Left] & \begin{matrix} [Hosp] & [OPD] & [NE] \end{matrix} \end{matrix}$  $Total cycles$  $\begin{matrix} [Hosp] & [OPD] & [NE] \end{matrix}$  $\left[ \begin{matrix} 1 & 0 & 0 & 0 & 0 \\ 0 & 1 & 0 & 0 & 0 \\ 0.50 & 0.50 & 0 & 0 & 0 \\ 0.54 & 0.46 & 0 & 0 & 0 \\ 0.59 & 0.41 & 0 & 0 & 0 \end{matrix} \right]$  $\left[ \begin{matrix} 2.76 & 1.49 & 4.77 \\ 1.92 & 2.8 & 5.76 \\ 2.03 & 1.78 & 6.89 \end{matrix} \right]= \left[ \begin{matrix} 9.02 \\ 10.48 \\ 10.70 \end{matrix} \right]$  $\bar{P}_{m}=\begin{matrix} \begin{matrix} [Dead] \\ \left[ Left \right] \end{matrix} \\ \begin{matrix} [Hosp] \\ \begin{matrix} \left[ OPD \right] \\ \left[ NE \right] \end{matrix} \end{matrix} \end{matrix}$  $F_{m}= \begin{matrix} \begin{matrix} [Hosp] \\ \begin{matrix} \left[ OPD \right] \\ \left[ NE \right] \end{matrix} \end{matrix} \end{matrix}$ | (i) |
| --- | --- | --- |

|  | $\begin{matrix} [Dead] & [Left] & \begin{matrix} [Hosp] & [OPD] & [NE] \end{matrix} \end{matrix}$  $Total cycles$  $\begin{matrix} [Hosp] & [OPD] & [NE] \end{matrix}$  $\left[ \begin{matrix} 1 & 0 & 0 & 0 & 0 \\ 0 & 1 & 0 & 0 & 0 \\ 0.33 & 0.67 & 0 & 0 & 0 \\ 0.34 & 0.66 & 0 & 0 & 0 \\ 0.39 & 0.61 & 0 & 0 & 0 \end{matrix} \right]$  $\bar{P}_{f}=\begin{matrix} \begin{matrix} [Dead] \\ \left[ Left \right] \end{matrix} \\ \begin{matrix} [Hosp] \\ \begin{matrix} \left[ OPD \right] \\ \left[ NE \right] \end{matrix} \end{matrix} \end{matrix}$  $\left[ \begin{matrix} 2.56 & 1.1 & 4.33 \\ 1.75 & 2.45 & 5.58 \\ 2.06 & 1.45 & 7.39 \end{matrix} \right]= \left[ \begin{matrix} 7.99 \\ 9.78 \\ 10.90 \end{matrix} \right]$  $F_{f}= \begin{matrix} \begin{matrix} [Hosp] \\ \begin{matrix} \left[ OPD \right] \\ \left[ NE \right] \end{matrix} \end{matrix} \end{matrix}$ | (ii) | |  |
| --- | --- | --- | --- | --- |
|  | $Total cycles$  $\begin{matrix} [Hosp] & [OPD] & [NE] \end{matrix}$  $\begin{matrix} [Dead] & [Left] & \begin{matrix} [Hosp] & [OPD] & [NE] \end{matrix} \end{matrix}$  $\left[ \begin{matrix} 2.57 & 1.21 & 4.49 \\ 1.75 & 2.54 & 5.86 \\ 1.92 & 1.52 & 7.07 \end{matrix} \right]= \left[ \begin{matrix} 8.27 \\ 10.15 \\ 10.51 \end{matrix} \right]$  $\bar{P}_{\geq65}=\begin{matrix} \begin{matrix} [Dead] \\ \left[ Left \right] \end{matrix} \\ \begin{matrix} [Hosp] \\ \begin{matrix} \left[ OPD \right] \\ \left[ NE \right] \end{matrix} \end{matrix} \end{matrix}$  $\left[ \begin{matrix} 1 & 0 & 0 & 0 & 0 \\ 0 & 1 & 0 & 0 & 0 \\ 0.47 & 0.53 & 0 & 0 & 0 \\ 0.50 & 0.50 & 0 & 0 & 0 \\ 0.56 & 0.44 & 0 & 0 & 0 \end{matrix} \right]$  $F_{\geq65}= \begin{matrix} \begin{matrix} [Hosp] \\ \begin{matrix} \left[ OPD \right] \\ \left[ NE \right] \end{matrix} \end{matrix} \end{matrix}$ | | (iii) | |

|  | $\begin{matrix} [Hosp] & [OPD] & [NE] \end{matrix}$  $\begin{matrix} [Dead] & [Left] & \begin{matrix} [Hosp] & [OPD] & [NE] \end{matrix} \end{matrix}$  $Total cycles$  $\left[ \begin{matrix} 3.35 & 1.94 & 5.87 \\ 2.53 & 3.25 & 6.83 \\ 2.85 & 2.39 & 8.42 \end{matrix} \right]= \left[ \begin{matrix} 11.16 \\ 12.61 \\ 13.66 \end{matrix} \right]$  $\bar{P}_{<65}=\begin{matrix} \begin{matrix} [Dead] \\ \left[ Left \right] \end{matrix} \\ \begin{matrix} [Hosp] \\ \begin{matrix} \left[ OPD \right] \\ \left[ NE \right] \end{matrix} \end{matrix} \end{matrix}$  $\left[ \begin{matrix} 1 & 0 & 0 & 0 & 0 \\ 0 & 1 & 0 & 0 & 0 \\ 0.22 & 0.78 & 0 & 0 & 0 \\ 0.25 & 0.75 & 0 & 0 & 0 \\ 0.28 & 0.72 & 0 & 0 & 0 \end{matrix} \right]$  $F_{<65}= \begin{matrix} \begin{matrix} [Hosp] \\ \begin{matrix} \left[ OPD \right] \\ \left[ NE \right] \end{matrix} \end{matrix} \end{matrix}$ | (iv) |
| --- | --- | --- |

| **Table 5a:** Predicted and Observed probabilities up to the 6th cycle (2 years) for male population | | | | | | | | | | | | | | | | | |
| --- | --- | --- | --- | --- | --- | --- | --- | --- | --- | --- | --- | --- | --- | --- | --- | --- | --- |
| **From** | **To** | **Prediction** | | | | | **Observed** | | | | | **Error** | | | | | |
|  | Cycle | ***[Dead]*** | ***[Left]*** | ***[Hosp]*** | ***[OPD]*** | ***[NE]*** | ***[Dead]*** | ***[Left]*** | ***[Hosp]*** | ***[OPD]*** | ***[NE]*** | ***[Dead]*** | ***[Left]*** | ***[Hosp]*** | ***[OPD]*** | ***[NE]*** |  |
|  |  |  |  |  |  |  |  |  |  |  |  |  |  |  |  |  |  |
| **BL** | **1** | - | - | - | - | - | 0.06 | 0.20 | 0.22 | 0.34 | 0.18 | - | - | - | - | - |  |
|  | **2** | - | - | - | - | - | 0.09 | 0.26 | 0.13 | 0.13 | 0.39 | - | - | - | - | - |  |
|  | **3** | 0.13 | 0.28 | 0.12 | 0.11 | 0.36 | 0.12 | 0.29 | 0.11 | 0.30 | 0.18 | 0.01 | 0.00 | 0.01 | -0.20 | 0.18 |  |
|  | **4** | 0.16 | 0.31 | 0.11 | 0.10 | 0.32 | 0.15 | 0.35 | 0.10 | 0.18 | 0.22 | 0.01 | -0.04 | 0.01 | -0.09 | 0.10 |  |
|  | **5** | 0.19 | 0.33 | 0.10 | 0.09 | 0.29 | 0.17 | 0.41 | 0.11 | 0.08 | 0.23 | 0.02 | -0.08 | 0.00 | 0.00 | 0.07 |  |
|  | **6** | 0.22 | 0.35 | 0.09 | 0.08 | 0.26 | 0.19 | 0.49 | 0.09 | 0.23 | 0.00 | 0.02 | -0.13 | 0.00 | -0.15 | 0.26 |  |
|  |  |  |  |  |  |  |  |  |  |  |  |  |  |  |  |  |  |
| The predicted probabilities derive from the model using only the observed data for the first two cycles. Note that because the model is constructed from the first two cycles, it makes no prediction for those cycles. *The left-hand columns show the predicted probabilities of the model, the columns at the centre represent the observed probabilities, and right-hand columns show the error (E) between two. For example, at cycle 4, the model predicts 16% patients will be dead, 31% have discharged, 11% patient will be hospitalised, 10% will be attended out-patient and 32% will not be required any HF service. Colour coding (heat map), as the difference increase changes from green to red. Negative signs indicate underestimation vice versa for positive sign. *Probabilities were rounded to 2 decimal points. Abbreviations: Hosp, hospitalised; OPD, out-patient clinic visit; NE, No event; BL, Baseline. | | | | | | | | | | | | | | | | | |

| **Table 5b:** Predicted and Observed probabilities up to the 6th cycle (2 years) for female population | | | | | | | | | | | | | | | | |
| --- | --- | --- | --- | --- | --- | --- | --- | --- | --- | --- | --- | --- | --- | --- | --- | --- |
| **From** | **To** | **Prediction** | | | | | **Observed** | | | | | **Error** | | | | |
|  | Cycle | ***[Dead]*** | ***[Left]*** | ***[Hosp]*** | ***[OPD]*** | ***[NE]*** | ***[Dead]*** | ***[Left]*** | ***[Hosp]*** | ***[OPD]*** | ***[NE]*** | ***[Dead]*** | ***[Left]*** | ***[Hosp]*** | ***[OPD]*** | ***[NE]*** |
|  |  |  |  |  |  |  |  |  |  |  |  |  |  |  |  |  |
| **BL** | **1** | - | - | - | - | - | 0.05 | 0.31 | 0.19 | 0.24 | 0.21 | - | - | - | - | - |
|  | **2** | - | - | - | - | - | 0.08 | 0.38 | 0.11 | 0.11 | 0.33 | - | - | - | - | - |
|  | **3** | 0.09 | 0.42 | 0.10 | 0.08 | 0.31 | 0.11 | 0.41 | 0.11 | 0.19 | 0.17 | -0.01 | 0.00 | -0.01 | -0.12 | 0.14 |
|  | **4** | 0.11 | 0.45 | 0.09 | 0.07 | 0.28 | 0.12 | 0.49 | 0.09 | 0.11 | 0.19 | -0.01 | -0.04 | 0.00 | -0.04 | 0.10 |
|  | **5** | 0.13 | 0.47 | 0.08 | 0.06 | 0.26 | 0.15 | 0.55 | 0.08 | 0.05 | 0.17 | -0.02 | -0.07 | 0.00 | 0.01 | 0.09 |
|  | **6** | 0.14 | 0.50 | 0.08 | 0.05 | 0.23 | 0.17 | 0.61 | 0.07 | 0.14 | 0.00 | -0.02 | -0.12 | 0.00 | -0.09 | 0.23 |
|  |  |  |  |  |  |  |  |  |  |  |  |  |  |  |  |  |

| The predicted probabilities derive from the model using only the observed data for the first two cycles. Note that because the model is constructed from the first two cycles, it makes no prediction for those cycles. *The left-hand columns show the predicted probabilities of the model, the columns at the centre represent the observed probabilities, and right-hand columns show the error (E) between two. For example, at cycle 4, the model predicts 11% patients will be dead, 45% have discharged, 09% patient will be hospitalised, 07% will be attended out-patient and 28% will not be required any HF service. Colour coding (heat map), as the difference increase changes from green to red. Negative signs indicate underestimation vice versa for positive sign. *Probabilities were rounded to 2 decimal points. Abbreviations: Hosp, hospitalised; OPD, out-patient clinic visit; NE, No event; BL, Baseline. |
| --- |

|  |
| --- |

| **Table 5c:** Predicted and Observed up to the 6th cycle (2 years) for ≥ 65 (years) population | | | | | | | | | | | | | | | | |
| --- | --- | --- | --- | --- | --- | --- | --- | --- | --- | --- | --- | --- | --- | --- | --- | --- |
| **From** | **To** | **Prediction** | | | | | **Observed** | | | | | **Error** | | | | |
|  | Cycle | ***[Dead]*** | ***[Left]*** | ***[Hosp]*** | ***[OPD]*** | ***[NE]*** | ***[Dead]*** | ***[Left]*** | ***[Hosp]*** | ***[OPD]*** | ***[NE]*** | ***[Dead]*** | ***[Left]*** | ***[Hosp]*** | ***[OPD]*** | ***[NE]*** |
|  |  |  |  |  |  |  |  |  |  |  |  |  |  |  |  |  |
| **BL** | **1** | - | - | - | - | - | 0.07 | 0.22 | 0.21 | 0.30 | 0.20 | - | - | - | - | - |
|  | **2** | - | -- | - | - | - | 0.10 | 0.28 | 0.12 | 0.12 | 0.37 | - | - | - | - | - |
|  | **3** | 0.14 | 0.31 | 0.11 | 0.09 | 0.35 | 0.13 | 0.32 | 0.11 | 0.25 | 0.18 | 0.00 | -0.01 | 0.00 | -0.16 | 0.17 |
|  | **4** | 0.17 | 0.34 | 0.10 | 0.08 | 0.32 | 0.16 | 0.39 | 0.10 | 0.15 | 0.21 | 0.01 | -0.05 | 0.00 | -0.07 | 0.11 |
|  | **5** | 0.19 | 0.36 | 0.09 | 0.07 | 0.28 | 0.19 | 0.45 | 0.10 | 0.07 | 0.20 | 0.00 | -0.09 | -0.01 | 0.01 | 0.09 |
|  | **6** | 0.22 | 0.38 | 0.08 | 0.07 | 0.26 | 0.22 | 0.52 | 0.08 | 0.18 | 0.00 | 0.00 | -0.14 | 0.00 | -0.12 | 0.26 |
|  |  |  |  |  |  |  |  |  |  |  |  |  |  |  |  |  |
| The predicted probabilities derive from the model using only the observed data for the first two cycles. Note that because the model is constructed from the first two cycles, it makes no prediction for those cycles. *The left-hand columns show the predicted probabilities of the model, the columns at the centre represent the observed probabilities, and right-hand columns show the error (E) between two. For example, at cycle 4, the model predicts 17% patients will be dead, 34% have discharged, 10% patient will be hospitalised, 08% will be attended out-patient and 32% will not be required any HF service. Colour coding (heat map), as the difference increase changes from green to red. Negative signs indicate underestimation vice versa for positive sign. *Probabilities were rounded to 2 decimal points. Abbreviations: Hosp, hospitalised; OPD, out-patient clinic visit; NE, No event; BL, Baseline. | | | | | | | | | | | | | | | | |

| **Table 5d:** Predicted and Observed up to the 6th cycle (2 years) for < 65 (years) population | | | | | | | | | | | | | | | | | |
| --- | --- | --- | --- | --- | --- | --- | --- | --- | --- | --- | --- | --- | --- | --- | --- | --- | --- |
| **From** | **To** | **Prediction** | | | | | **Observed** | | | | | **Error** | | | | | |
|  | Cycle | ***[Dead]*** | ***[Left]*** | ***[Hosp]*** | ***[OPD]*** | ***[NE]*** | ***[Dead]*** | ***[Left]*** | ***[Hosp]*** | ***[OPD]*** | ***[NE]*** | ***[Dead]*** | ***[Left]*** | ***[Hosp]*** | ***[OPD]*** | ***[NE]*** |  |
|  |  |  |  |  |  |  |  |  |  |  |  |  |  |  |  |  |  |
| **BL** | **1** | - | - | - | - | - | 0.02 | 0.31 | 0.20 | 0.32 | 0.15 | - | - | - | - | - |  |
|  | **2** | - | - | - | - | - | 0.04 | 0.38 | 0.12 | 0.12 | 0.34 | - | - | - | - | - |  |
|  | **3** | 0.05 | 0.41 | 0.12 | 0.10 | 0.32 | 0.05 | 0.41 | 0.11 | 0.28 | 0.15 | 0.00 | -0.01 | 0.02 | -0.18 | 0.17 |  |
|  | **4** | 0.06 | 0.44 | 0.11 | 0.10 | 0.30 | 0.06 | 0.47 | 0.09 | 0.18 | 0.20 | 0.00 | -0.03 | 0.02 | -0.08 | 0.09 |  |
|  | **5** | 0.07 | 0.47 | 0.10 | 0.09 | 0.27 | 0.07 | 0.54 | 0.09 | 0.08 | 0.22 | 0.00 | -0.07 | 0.02 | 0.00 | 0.05 |  |
|  | **6** | 0.08 | 0.50 | 0.10 | 0.08 | 0.25 | 0.08 | 0.61 | 0.08 | 0.24 | 0.00 | 0.00 | -0.11 | 0.01 | -0.15 | 0.25 |  |
|  |  |  |  |  |  |  |  |  |  |  |  |  |  |  |  |  |  |
| The predicted probabilities derive from the model using only the observed data for the first two cycles. Note that because the model is constructed from the first two cycles, it makes no prediction for those cycles. *The left-hand columns show the predicted probabilities of the model, the columns at the centre represent the observed probabilities, and right-hand columns show the error (E) between two. For example, at cycle 4, the model predicts 06% patients will be dead, 44% have discharged, 11% patient will be hospitalised, 10% will be attended out-patient and 30% will not be required any HF service. Colour coding (heat map), as the difference increase changes from green to red. Negative signs indicate underestimation vice versa for positive sign. *Probabilities were rounded to 2 decimal points. Abbreviations: Hosp, hospitalised; OPD, out-patient clinic visit; NE, No event; BL, Baseline. | | | | | | | | | | | | | | | | | |
